# Teacher Cognition and Practices in Using Generative AI Tools to Support Student Engagement in EFL Higher-Education Contexts

**DOI:** 10.3390/bs15091202

**Published:** 2025-09-04

**Authors:** Senem Zaimoğlu, Aysun Dağtaş

**Affiliations:** Department of English Translation and Interpreting, Çağ University, Mersin 33800, Türkiye; aysunyurdaisik@cag.edu.tr

**Keywords:** teacher cognition, Generative AI, narrative inquiry, student engagement, EFL teaching

## Abstract

As Generative Artificial Intelligence (GenAI) tools become increasingly embedded in higher education, English as a Foreign Language (EFL) teachers are challenged to rethink their pedagogical identities and practices. While policy discourses often promote GenAI for its potential to enhance instructional efficiency, little is known about how language teachers conceptualize and employ these tools to foster meaningful student engagement. This study explores how university-level EFL teacherinterpret and implement GenAI technologies to support student engagement through narrative inquiry. Drawing on three-level narrative approach, story, Story, and STORY, it examines nine language teachers’ retrospective experiences with GenAI tools across personal, contextual, and sociopolitical dimensions. The findings indicate that teachers’ interactions with GenAI are shaped by their pedagogical affordance, as well as their teaching values, imposed political agendas, and professional agency. This study calls for professional development programs specifically designed for teachers’ narrative identities to ultimately facilitate the ethical use of GenAI in learner-centered EFL contexts.

## 1. Introduction

The recent surge in Generative Artificial Intelligence (GenAI) tools (e.g., ChatGPT [GPT-4, OpenAI], Bard [Google, Gemini] and Claude [Anthropic, v3]) has radically transformed education in the last several years, leading educators to rethink long-held values and practices (Giannakos et al., 2024; Harry, 2023). This is especially highlighted in English as a Foreign Language (EFL) education, which is centered around communicative competence and human interaction, as GenAI tools present an opportunity and a challenge to support existing teaching practices, create content, and help learners develop language skills (Giannakos et al., 2024; Harry, 2023). Indeed, language learners could complete many existing tasks in an efficient manner, such as grammar checking, writing facilitation, and vocabulary development (Ng et al., 2021). In addition, they are used to create dynamic environments for language learning. They can engage learners in dynamic dialogue, take on real-world uses of language, and allow students to use language in context (Zhang & Dong, 2024). This kind of talk-as-practice is ideal in second language learning as it supports formulating fluency and confidence in a non-pressured environment (Giannakos et al., 2024). For instance, some language-learning platforms have included GenAI-powered chatbots that can mimic the conversational abilities of native speakers to allow learners to practice the language in an interactive, conversational way (Chen et al., 2025). Another significant application of GenAI tools in language education is content generation. With these tools, teachers can generate their own learning materials, including reading texts, as well as quizzes and exercises, for language learners of different levels (Moorhouse & Wong, 2025). This capability quickly produces relevant content, which means that teachers can spend less time on the administrative work of creating materials and more time on their pedagogical practice. This case is also true for adaptive learning platforms, where learners use these tools to provide exercises to students with adaptive manipulation based on the students’ performance, where learners will always be challenged without being pushed (Contrino et al., 2024). However, the rise of GenAI tools in the classroom raises additional questions such as their accuracy, reliability, and ethical usage (Nguyen et al., 2024).

### 1.1. Challenges in the Use of Generative AI

Teachers play a very important role in deciding how these GenAI tools are integrated into the classroom setting and evaluating the possible benefits and challenges (de Fine Licht, 2024; Mabuan, 2024). One problem that teachers perhaps often complain about is the accuracy and reliability of GenAI-generated content. In some situations, they may provide grammar corrections or language suggestions, but at other times, they may generate sentences with inappropriate or incorrect grammar phrasings that confuse students (Harry, 2023). Moreover, they may create culturally or contextually inappropriate examples that have the potential for misunderstandings or reinforce stereotypes in language learning resources (Warschauer & Xu, 2024). Another serious concern is the over-reliance on language technology tools that can diminish students’ abilities to develop independence regarding their language skills, as well as separate abilities to develop critical thinking strategies (Chiu et al., 2023). For example, if students use GenAI tools to write an essay or to respond to a question, they may skip the critical thinking and problem-solving process (Hong, 2023). Rahimi (2025) notes the importance of critical thinking and communication skills when interacting with Generative AI tools, stating that these skills are necessary to identify and address potential issues such as bias, misinformation, and ethical misuse. Teachers need to balance the benefits offered by new technologies with an acknowledgement of such challenges, with a view to utilizing GenAI tools in providing greater leverage for students’ learning while minimizing identified risks (Jin et al., 2025)

Taking all these issues into account, the integration of GenAI into language education holds both a great deal of promise and some major drawbacks. However, considering the efficiency, personalization, and innovation that GenAI can bring to language instruction, relatively little is known about how language teachers conceptualize and engage with these technologies in practice—especially in relation to fostering student engagement.

### 1.2. Fostering Student Engagement Through Generative AI

Student engagement, which has become a key indicator of the quality of student learning and academic motivation, is widely recognized as a multidimensional construct encompassing three dimensions: behavioral, emotional, and cognitive (Fredricks et al., 2004). Each dimension contributes uniquely to the learning process, yet they operate in dynamic interplay within the classroom environment. Moreover, these dimensions are increasingly shaped by the pedagogical affordances of emerging technologies, particularly in digitally mediated learning environments influenced by GenAI.

Behavioral engagement, for instance, refers to students’ observable participation in academic activities. This includes regular classroom attendance, focus on instruction, completion of a task in a given time, and willingness to participate in class discussions (Appleton et al., 2008). In EFL classes, these aspects of EFL learner engagement can be influenced by the accessibility and reactivity of GenAI tools due to the fact that they encourage learners to engage with language-related tasks more frequently and confidently (Philp & Duchesne, 2016). Recent research suggests that GenAI tools have the potential to enhance this dimension by providing immediate, personalized feedback in a manner that enhances the student’s perseverance toward the completion of tasks (Kasneci et al., 2023). For example, the use of AI writing assistants, or conversational tools, allows students to rehearse linguistic structures or find other ways of phrasing things without the fear of making a mistake in public, which helps build students’ confidence and their participation in class.

Conversely, cognitive engagement is defined as the psychological investment that students make when creating an understanding of, and becoming competent with, academic material, and this includes deep learning strategies that include elaboration, organization, and critical thinking, as well as metacognitive strategies for monitoring, planning, and evaluating one’s learning activities (Alam & Mohanty, 2024). Cognitively engaged language learners are more inclined to approach a task with a curious mindset, question grammatical rules underlying their language learning, and employ self-regulatory strategies to deepen their understanding (Vo et al., 2025).

GenAI tools, when used for pedagogical aims, can act as cognitive scaffolds, either by providing models of complex language use, by engaging learners in reflection, or by helping learners to evaluate and refine their own outputs (Zawacki-Richter et al., 2019). For example, by having students reflect on multiple revisions or rationales for their language choices, GenAI-assisted tools can facilitate the use of higher-order thinking and strategy use, which are also indicators of deep cognitive engagement. Moreover, emotional engagement is a dimension that describes the affective engagement of students in learning contexts (Pekrun, 2006). This dimension is especially pertinent in EFL contexts, where students may face disturbing conditions in language performance, such as fear of negative evaluation and speaking in front of their peers (Dewaele & MacIntyre, 2016). GenAI tools could provide conditions for practice that enable emotional engagement through psychologically safe, informal, and judgment-free experiences. For example, learners prefer using GenAI tools more as they provide less stressful conditions and dispel their anxiety, particularly in language performance (Qin et al., 2020). Furthermore, the novelty and personalization afforded by GenAI applications could also further engage the curiosity and emotional connection to tasks crucial for motivation and engagement (Hu & Shao, 2025). However, emotional engagement with GenAI applications is not always positive; excessive reliance on GenAI applications to produce pedagogically valuable results, ethical ambiguities, or inauthentic feedback could result in confusion or distrust (Crompton et al., 2024), which points to the need for some pedagogical mediation.

It is apparent that all dimensions of engagement are not only outcomes but also potential mediators of successful language teaching. As Reeve (2012) points out, teaching practices that promote autonomy, competence, and relatedness enhance all three dimensions of engagement. Therefore, any educational innovation, including the implementation of GenAI tools, should be evaluated in terms of both the quality and integrity of application, as well as the qualities of holistic engagement for students (Belkina et al., 2025). This is a salient consideration for those working in EFL contexts, where engagement is highly dependent on many other intersecting factors. However, little is known about the impact of GenAI on these multiple layers of engagement. Understanding this landscape is crucial to developing high-quality, ethically sound, learner-centered, and context-responsive uses for AI in language education.

### 1.3. The Current Study

The process of integrating GenAI tools into educational practice is more than simply a technical act. It is a pedagogical, emotional, and identity-based act that must acknowledge that teachers are not merely recipients of innovation. Each teacher makes active and value-laden decisions about certain technologies in ways that address their instructional objectives, student needs and motivations, and their own value-laden beliefs about teaching and learning (Prestridge, 2012). In Borg’s (2006) Teacher Cognition Theory, he emphasized that a teacher’s knowledge, beliefs, and feelings, shaped by their previous experiences, training, and classroom contexts, are fundamental in understanding their pedagogical decisions. Fives and Buehl (2012) state that these processes are shaped by their institution, curriculum, and socio-cultural contexts. Apart from Borg’s theory, Self-Determination Theory (Deci & Ryan, 2000), and Control–Value Theory (CVT) (Pekrun, 2006; Pekrun & Perry, 2014) have also been taken into account. These theories illustrate how perceptions of autonomy, competence, and task value impact not only teachers’ willingness to engage with GenAI tools but also the emotional climates they create in their classrooms. Teachers who perceive institutional support and feel free in their exploration of GenAI expressed themselves, as they feel more excited and have a greater sense of agency, a development that aligns with SDT’s focus on the necessity to satisfy basic psychological needs (Deci & Ryan, 2008; Reeve, 2012). Conversely, top-down use of GenAI without sufficient training or dialogue can lead to feelings of anxiety, resistance, or disconnection—emotions that are consistent with CVT. Pekrun and Linnenbrink-Garcia (2012) note that negative emotions triggered by educational tasks occur when people experience low control or place low value on these tasks. Moreover, Ertmer and Ottenbreit-Leftwich (2010) asserted that the adoption of technology is mediated through teachers’ pedagogical beliefs and confidence and the culture of the school. It could also be argued that Gen AI adoption is more than access or the ability to use technology; it is a process intimately tied to teachers’ epistemological beliefs, perceptions of professional agency, and even widely held beliefs about educational values. Thus, the adoption of GenAI cannot be conceptualized solely as an access and ability to use technology, but as a process that is intrinsic to teachers’ epistemological beliefs, perceptions of professional agency, and values about education more generally (Cabellos et al., 2024). For instance, educators who see GenAI as a mechanism to enhance learner autonomy and creativity may commit themselves to it and consider it a colleague in the classroom. In contrast, teachers who perceive GenAI tools as risky or view them as unethical or imperfect cognitive processes may be on the fence about using them. This hesitation will exist despite policies that are committed to implementing such tools (Barrett & Pack, 2023). In short, properly understanding how language teachers use GenAI will require understanding the implicit and often unspoken elements of professional identity and decision-making in technology-enhanced language education.

From this perspective, incorporating GenAI is not just a pedagogical choice about teaching practices, but a kind of identity work. It shows how teachers balance their moral obligations, professional agency, and changing professional identities in an increasingly technological world. As Selwyn (2011) explained, educational technologies are not neutral; they are always intertwined in social practices and power relations. Consequently, any attempt to promote the meaningful integration of GenAI into language education must move beyond tool improvement to the cognitive, affective, and behavioral dimensions of teacher development, which means that teachers are not passive recipients of technological change but reflective practitioners shaping the ethical and pedagogical futures of their classrooms (Walter, 2024). Although teachers are given great importance in language education, most empirical studies have still focused on student outcomes or ethical concerns (Luckin et al., 2016; Selwyn, 2011), with limited attention to how language teachers experience and implement GenAI in real classroom settings. Even fewer have explored this from a narrative and engagement-centered perspective. Therefore, this study seeks to fill this gap by examining how university-level EFL teachers interpret and utilize GenAI tools to support student engagement. In line with this, the current study tries to explore the following research questions.

1. How do university-level EFL teacherdescribe their classroom uses of GenAI to foster students’ behavioral, cognitive, and emotional engagement?
2. In what ways do teacher’ beliefs, roles, and program/institutional conditions shape their pedagogical decisions about integrating GenAI?
3. How do higher-education policies, ethical considerations, access, and digital infrastructure enable or constrain GenAI implementation for student engagement?

## 2. Materials and Methods

### 2.1. Research Design

**Figure 1:**
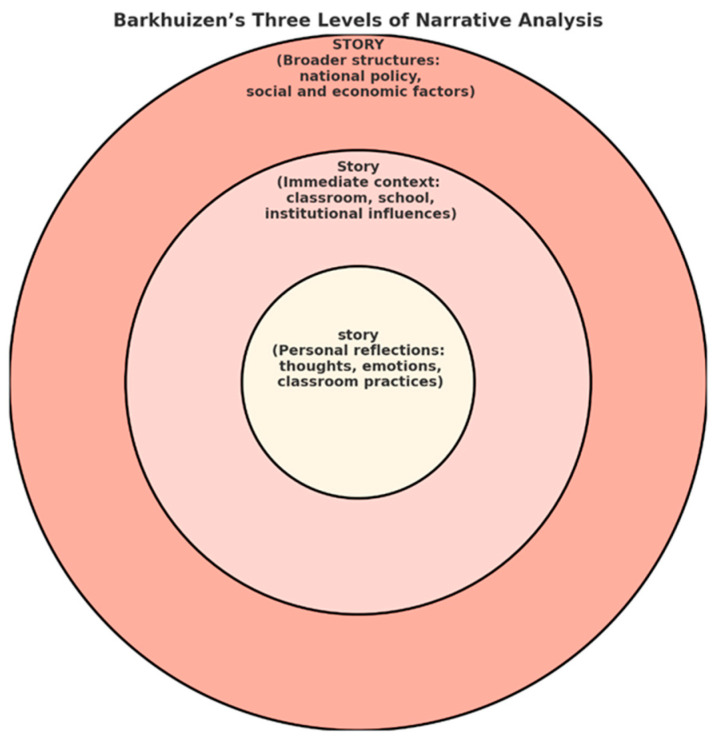
Barkhuizen’s (2016) model of narrative analysis.

This study adopts a qualitative narrative inquiry approach to explore how university-level EFL teacher conceptualize and implement GenAI tools in ways that support student engagement. The research design is guided by Barkhuizen’s (2016) model of narrative analysis, which conceptualizes narrative meaning-making through three interrelated levels—the personal narrative (story), which encompasses individual thoughts, emotions, and ideas; the educational context (Story), including the school, classroom, or natural language learning environment; and the broader societal context (STORY), which involves national language learning policies, curricula established by the Council of Higher Education, and the socioeconomic conditions of the region (shown in Figure 1). These levels are examined through three dimensions: content, which focuses on characters and their relationships; setting, which pertains to locations and sequences; and temporal aspects, which address time frames.

Barkhuizen (2007) asserts that the initial level of narration, referred to as the story, constitutes the inner circle. At the story level, university-level EFL teachernarrate their personal and situated experiences with GenAI as they attempt to support student engagement. These narratives includeteacher’ reflections on specific classroom episodes—such as using AI chatbots to stimulate student curiosity, implementing GenAI tools in collaborative writing tasks to boost participation, or navigating moments of hesitation regarding students’ ethical use of AI. The story level capturesteacher’ immediate pedagogical interactions and emotional responses as they experiment with GenAI tools to enhance student motivation, critical thinking, or classroom presence. It also includes the social interactions that take place in these contexts, such as conversations with students about their experiences using GenAI, informal feedback shared after class, or personal reflections documented in teaching journals. In this inner circle, the temporal and spatial scales are restricted, and this allows for an emphasis on intimate, micro-level interactions and personal meaning for teachers using GenAI in their teaching practice to enhance student engagement (Barkhuizen, 2016). The second level, the Story level, connects teachers’ classroom experiences with GenAI to broader narratives as professionals and institutions. At this level, participants contemplate their own beliefs about teaching, learning, and educational technology and their collective pedagogical practices and beliefs about GenAI through narratives. These narratives reflect shifting views of classroom realities—how GenAI may alter the ways students are motivated, participate, or become emotionally engaged in their learning—and how these shifts may be impacting their identities as language teachers within a widely disruptive social shift toward digital transformation. This level also encompasses influences that extend beyond the individual classroom, e.g., institutional policies, curriculum expectations, the expectations of programs, and wider discourses about AI and education. Teachers describe how institutional policies constrain or facilitate students’ use of GenAI; for example, how they may or may not foster experimentation with GenAI tools, whether they provide training or impose some ethical policies shaping ideas about how students can use GenAI. In addition, following Barkhuizen’s (2007) model, this level is also concerned with group dynamics, classroom interactions, teacher and learner competencies, and the socio-material context. As teachers strive to use GenAI to engage students, they are frequently confronted by factors that are beyond their control—the types of digital literacies their students possess, classroom technology and instructional practices, and even their students’ general openness to technology. These broader elements frame the ways in which GenAI is implemented, perceived, and adapted to support or facilitate student engagement within a culture.

The STORY level expands our understanding to the macro-level of analysis involving the sociopolitical and institutional contexts that underpin teachers’ classroom practices using GenAI. At this level, the narratives of participants reveal how their decisions to use GenAI to support student engagement are not just dependent on their pedagogical intentions but are also influenced by more systemic factors such as higher education policies, institutional policies, and wider literacies regarding technology and artificial intelligence in higher education. Teachers often rationalize their experiences based on being a part of larger discussions about the digitization of education, questions about ethics pertaining to algorithmic bias and surveillance, equity of student access to GenAI tools, and concerns regarding issues of academic integrity. Additionally, their decisions are subject to Council of Higher Economic Development standards and curriculum decisions, as well as the socioeconomic realities of their students, including student digital literacy challenges or access to technological infrastructure. These macro-level forces could either enable or constrain how and if teachers saw GenAI as a way to support students cognitively (e.g., by providing personalized feedback), emotionally (e.g., harnessing student motivation and curiosity), or behaviorally (e.g., facilitating active engagement).

As Barkhuizen (2007) notes, at this level, teaching is not conducted in a vacuum; rather, it is informed by ideological, cultural, and political structures. Institutions and policy directives frame the acceptable boundaries of technology use in the classroom, shaping teacher autonomy and pedagogical innovation. Following Kumaravadivelu (2006), classroom practices are seen as dialogically shaped by larger social, political, and economic landscapes, in which teachers and students bring situated experiences and values. These macro-contexts reciprocally interact with micro-level teaching decisions, ultimately influencing how GenAI is conceptualized, negotiated, and enacted to support meaningful student engagement.

### 2.2. Research Site and Participants

A sample of 9 out of 34 university-level English teachers with different backgrounds at a foundation university in Türkiye was chosen to capture the wide range of opinions regarding the use of GenAI tools in language teaching (Table 1). Participants were selected based on their familiarity with these tools and their active use of these technologies in their teaching to support students’ engagement. To ensure that participants were both familiar with and actively using GenAI tools in their teaching, a brief pre-screening process was conducted prior to data collection. The study involved a brief questionnaire, in which potential participants answered questions about their knowledge and use of GenAI technologies (e.g., ChatGPT and AI-based writing assistants), how they had used these tools in their teaching, and to what extent they had used this technology to promote students’ engagement. Only those who confirmed both knowledge and practical experience with GenAI were invited to participate, ensuring that the narratives collected were based on real classroom experience with AI.

The selected participants had a range of academic backgrounds, from English Language Teaching (ELT) to Translation and Interpretation and English Literature. This diversity was intentional because research indicates that teachers’ backgrounds can influence their beliefs and practices regarding technology usage in the classroom (Ertmer & Ottenbreit-Leftwich, 2010). With respect to gender, a clear majority of the participants (N = 6) were female, which may have shaped the narratives provided, particularly in terms of emotional expression, teaching style, and perceptions about technology use. According to Ertmer and Ottenbreit-Leftwich (2010), gender shapes teachers’ beliefs regarding educational innovations and classroom interactions. On the teaching front, the participants’ level of experience ranged from 3 years to over 20 years. This diversity allowed for a deeper discussion on how experience shapes the understandings and ways teachers work with AI. It is important to consider both new and experienced teachers, as the literature suggests that one’s experience can make them less likely to accept new teaching tools (Kohnke et al., 2023). Furthermore, they also had varied levels of familiarity with GenAI tools, from low to high, which allowed for a more robust perspective on how the different levels of digital competence shape participants’ perceptions of integrating GenAI into their teaching. In sum, participant selection aimed to include a range of perspectives and experiences to better understand what it means to integrate AI technology into university-level English language teaching and learning.

Regarding our positionality as researchers in the study, it came from our backgrounds as educators and researchers of English as a Foreign Language (EFL) at a tertiary level and a developing scholarly interest in the pedagogical implications of GenAI tools. Our professional experience in EFL was advantageous because it allowed us to develop our positionality to engage in this phenomenon with an informed curiosity, but it also made our interpretive stance susceptible to some introjection. We understood that our engagement with GenAI was as both users and analysts of GenAI; therefore, our orientation in our interpretation of our participants’ narratives was formed through our own engagement with GenAI. Throughout the course of the research, we endeavored to maintain a reflective stance.

### 2.3. Data Collection

As the primary purpose of this study was to investigate university-level EFLteacher’ experiences with Generative AI (GenAI) to support their students’ engagement within social, institutional, and pedagogical contexts, they were asked to describe narratives of their own teaching experiences with GenAI tools. These written reflections were also intended to capture the tteachers’ perceptions, practices, and feelings in connection to their use of GenAI to enhance their students’ engagement in the classroom (Fredricks et al., 2004; Reeve & Tseng, 2011).

The participants were given a set of open-ended prompts that were based on Barkhuizen’s (2007, 2016) narrative framework to utilize before they began writing. The prompts were developed based on their classroom experiences, incorporating GenAI tools, student reactions, their motivation or hesitation, and other social or institutional elements shaping responses and decisions. For example, at the story level, teachers were asked to recall particular classroom examples when they used GenAI to support student engagement and to recall the emotions and teaching decisions associated with those occasions; at the Story level, participants were prompted to reflect on how various larger teaching philosophies, institutional roles, and environments informed their use of GenAI as a tool; and at the STORY level, they were asked to write whether national education policies, Higher Education Council (YÖK) regulations, or curriculum requirements impose limitations on their use of GenAI tools in teaching. The participants had two weeks to complete their narratives and submit them through an online form. The narratives ranged from approximately 500 to 1200 words in length. They were informed that there were no right or wrong answers and were encouraged to write freely. In addition, participants were able to choose to write in either English or Turkish, depending on their preference and comfort. While some participants submitted their narratives in English, participants who wrote in Turkish had their narratives translated by the researcher, ensuring the meaning and context were maintained. This allowed for an analysis of the data while preserving the authenticity of the participant’s voice. It also supported the collection of rich narratives of experience based on the realities of classroom practice, specifically illustrating how GenAI is either meaningfully integrated or resisted in the everyday practices of EFL teachers (Barkhuizen, 2016; Clandinin & Connelly, 2000).

### 2.4. Data Analysis

The qualitative data gathered through written narratives were analyzed with Shedivy’s (2004) four-step phenomenological approach, layered with Barkhuizen’s (2007, 2016) three-level narrative model (story, Story, and STORY). This approach allowed for multiple layers of understanding of participants’ lived experiences regarding Generative AI (GenAI) while remaining methodically and conceptually situated within both phenomenological meaning-making and a narrative framework. Firstly, each narrative was read a few times carefully to obtain a sense of the participant’s perspective in total. In the second step, significant statements related to teachers’ use of GenAI and its perceived impact on students engaged with GenAI were extracted from the narratives. These statements were then formulated into meanings that captured the intention, emotional response, and interpretations of the participants. In the third step, the formulated meanings were grouped into themes, paying careful attention to overlapping similarities and differences within cases. The identified themes most authentically represented participants’ pedagogical practice, emotional responses, and contextual factors regarding the integration of GenAI in their contexts. Finally, the themes were grouped, organized, and interpreted through Barkhuizen’s three narrative levels. At the story level, themes represented personal classroom pedagogical acts and emotional experiences engaging with GenAI tools; the Story level described general pedagogical beliefs, institutional context, and teacher identities; and the STORY level addressed broader sociopolitical considerations such as policy discourses, equity, and digital infrastructure. These “groupings” were organized and interpreted according to Clandinin and Connelly’s (2000) descriptions of interpretive narratives, maintaining a multi-layered complexity of how GenAI is experienced, interpreted, and implemented by university-level EFL teachers.

To enhance the trustworthiness of the analysis, illustrative quotes were carefully selected to support thematic interpretations. A second researcher conducted a review of the coding independently to verify the category in order to establish inter-rater agreement. A reflexive journal was used throughout the research process to log analytic decisions and the researchers’ positionality. Thematic analysis continued until saturation was reached and no new themes emerged from the data. Selected thematic interpretations were also shared with some participants for member validation, further reinforcing the credibility of the study. All analysis was completed manually and through Microsoft Word and Excel only. Key narrative excerpts were first highlighted in Word documents, with initial codes inserted as margin comments. These coded segments were then organized into an Excel matrix, where they were grouped by thematic similarity and aligned with Barkhuizen’s three-tiered framework. This approach allowed for the systematic tracking of codes, themes, and supporting quotes, thereby strengthening the transparency and rigor of the analysis. This layered and transparent analytical process enabled a nuanced understanding of how EFL teachers navigate GenAI use across intersecting micro-, meso-, and macro-educational contexts.

### 2.5. Ethical Considerations

Ethical approval for this study was obtained from the relevant institutional review board at Cag University. All participants provided informed consent after being informed about the purpose, procedures, and potential uses of the study. Participation was entirely voluntary, and participants were made aware of their right to withdraw at any time. Pseudonyms were used to protect participant identity, and all data were treated with strict confidentiality. The intention to publish the results was clearly stated prior to data collection.

## 3. Results

### 3.1. Participants’ story, Story, and STORY Analyses

This section presents illustrative excerpts from the participants’ narratives to exemplify the three interrelated levels of analysis—story, Story, and STORY—as conceptualized and highlighted in our narrative inquiry framework and research questions.

While the narrative format captures the richness of participants’ voices, we also present a brief summary in Table 2, which details the themes at each narrative level and demonstrates them through one or two representative participant quotes, making it easier to compare themes across levels before giving detailed information for each level.

#### 3.1.1. story

Within the framework of the first level—the story level—participants shared classroom-based reflections that revealed how they used GenAI tools to actively support student engagement. These narratives highlighted micro-level teaching practices, such as real-time classroom activities and individual student–teacher interactions, through which teachers experimented with GenAI to enhance participation, motivation, and critical thinking.

For instance, Participant 3 described using an AI chatbot in a writing class to assist students in brainstorming ideas for argumentative essays. Her practice was intentionally designed to stimulate cognitive engagement through idea generation while also encouraging behavioral participation and emotional involvement. She observed that the use of GenAI unexpectedly empowered quieter students to contribute, enhancing both their confidence and collaborative learning dynamics.

*“One of my quieter students even raised her hand to share what the chatbot suggested and how she disagreed with it. That was a moment I didn’t expect—it felt like the tool had given her a voice. I felt cautiously optimistic about using it again.”*

Similarly, Participant 2 recounted how she used GenAI in a speaking-focused activity to help students rehearse for oral presentations. Initially hesitant about its pedagogical value, she later observed that the AI-supported practice allowed a typically disengaged student to confidently paraphrase content and initiate peer discussion. The activity promoted deeper cognitive processing of ideas, boosted the student’s emotional self-assurance, and prompted active behavioral participation.

*“I was unsure about using ChatGPT at first. But I tried it in a prep activity for oral presentations… One of my quieter students read what the AI suggested and then rephrased it in her own words during the rehearsal. That was the first time I saw her take the lead in a speaking task. It changed how I viewed the tool—it can actually bring some students out of their shells.”*

In reading-focused activities, Participants 5 and 6 asked students to use GenAI tools to summarize articles before class discussions. This lowered cognitive barriers and encouraged participation. As one teacher noted, *“Students who usually stay quiet started jumping into the discussion… the room felt more alive*.”

Participant 8 used a GenAI tool to provide formative feedback on students’ draft essays before final submission. Instead of traditional margin comments, she had students input their drafts into the AI to identify grammar issues, vague arguments, or coherence problems.

*“I asked students to use Grammarly to review their first drafts before handing them in. One student told me, ‘It’s like having a personal editor.’”*

These classroom practices illustrate how GenAI can serve not merely as a technical support tool but as a pedagogical mediator that enables teachers to foster multidimensional engagement—especially among students who might otherwise remain passive or withdrawn.

#### 3.1.2. Story

At the second level—Story (with a capital S)—participants contextualized their use of GenAI within broader institutional and pedagogical frameworks, highlighting how their practices were shaped by school policies, departmental culture, or administrative decisions. These reflections revealed how teaching with GenAI was not only a personal or classroom-based act but also embedded within a web of professional responsibilities and institutional expectations. For instance, Participant 4 described how a university-led initiative encouraging digital tool integration pushed him to explore GenAI platforms more systematically in his curriculum. He noted that the institutional support—through workshops and open conversations with colleagues—made him feel more confident and willing to try AI-supported speaking activities, particularly in courses that previously relied on traditional oral tasks. This institutional backing, he reflected, transformed his initial reluctance into a sense of empowerment.

*“When our department started encouraging experimentation with GenAI tools, I felt more supported. I wasn’t the only one trying something new, and that made a big difference.”*

Similarly, Participant 9 shared how her department initiated a collaborative workshop series where teachers exchanged strategies for integrating GenAI into their EFL classrooms. Through these discussions, she felt encouraged to experiment with GenAI tools during group projects, especially in reading and vocabulary tasks. This support network not only boosted her confidence but also helped her refine classroom practices that engaged students on multiple levels.

*“After attending a departmental session on AI in language teaching, I realized I wasn’t alone in feeling uncertain. A colleague shared how she used GenAI tools for vocabulary enrichment, and I adapted the idea.”*

Other participants described navigating institutional constraints. Participant 7 recounted how GenAI was not officially endorsed due to plagiarism concerns, but he incorporated it informally in pre-writing tasks to help students brainstorm ideas: *“We weren’t allowed to assign AI writing tools directly, but I still wanted my students to explore them responsibly.”* Participant 2 also described how new institutional guidelines on ethical AI use in education led her to rethink her classroom activities. Concerned about academic integrity, she designed a project that integrated GenAI into writing lessons but framed the tool explicitly as a brainstorming and drafting aid rather than a substitute for original work. She noted students’ increased sense of responsibility and openness in reflecting on their use of AI.

*“After our university guidance on AI ethics, I redesigned my writing tasks. Students had to write reflective journals about how they used ChatGPT—what they accepted, what they changed, and why. It was a way to keep things transparent but also critical.”*

The participants also reflected on policy-driven transformations. Participant 1 admitted he began using GenAI mainly because of institutional development goals but later came to value its pedagogical benefits, particularly in peer-review sessions: *“Honestly, I started using AI because it was part of our institutional goals… but I realized this wasn’t just about tech—it was about agency.”* Likewise, Participant 6 described how a digitalization policy and professional development program prompted him to experiment with GenAI in reading classes, which increased student comprehension and sparked discussions about AI bias. After incorporating GenAI to summarize and paraphrase complex texts, he observed increased student motivation and improved comprehension.

*“Our faculty encouraged us to explore GenAI through a professional development program. I was skeptical at first, but I tried it with a reading class. I asked students to use Quillbot to paraphrase difficult passages, and it worked surprisingly well. It not only helped them understand the content better, but it also sparked conversations about language use and bias in AI. That level of engagement rarely happens with textbook readings.”*

Together, these narratives illustrate how institutional structures both enabled and constrained GenAI integration. Teachers’ reflections show that institutional initiatives, policies, and collegial support networks influenced not only their practices but also their evolving professional identities.

#### 3.1.3. STORY

The third and outermost level of Barkhuizen’s (2007) narrative framework—STORY—encapsulates the macro-level sociopolitical forces that shape how teaching and learning occur. This level reflects how national educational policies, institutional mandates, and broader ideological discourses inform teachers’ classroom practices, often in ways that are beyond their immediate control. As Chankseliani et al. (2021) argue, educational decisions at national and local levels reflect prevailing political ideologies and worldviews. In language education, this includes curriculum design, access to technology, and definitions of “acceptable” pedagogical innovation. Liddicoat and Han (2024) remind us that English language teaching is always situated in larger cultural and ideological structures, shaped by globalization, policy, and linguistic hierarchies. Thus, teachers’ GenAI practices must be understood within these broader contexts.

In the current study, several participants referenced how Council of Higher Education guidelines, institutional expectations, and regional socioeconomic disparities shaped their ability to implement GenAI tools meaningfully. For example, three teachers reported that their early efforts to incorporate GenAI were constrained by outdated curriculum standards, a lack of infrastructure, or pressure to adhere to exam-oriented teaching goals. These systemic pressures often conflicted with their desire to promote deeper student engagement through innovative tools. Participants described being “cautious,” “limited,” or “unsure” due to a lack of national-level clarity on GenAI integration in education.

Participant 8 directly linked her evolving classroom practices to national discourse on digital transformation. While she was enthusiastic about using GenAI to support student creativity, she expressed concern that the national curriculum emphasized measurable outcomes and test preparation over exploratory learning.

*“There’s a push for using new technologies like AI, but the exams still dominate everything. I tried to use ChatGPT to let students explore debate topics more freely, but later I had to go back and prepare them for grammar-based questions for the proficiency test. It feels like two different worlds—one says innovate, the other says don’t take risks.”*

Participants also emphasized how institutional ambiguity and risk-averse ethical discourses generated hesitation around GenAI integration. While universities have promoted innovation in principle, explicit policies or guidelines are often absent. Participant 6 explained that while their university promoted digital literacy and innovation in public statements, there was no official policy or training on how to integrate GenAI tools.

*“There’s a lot of talk about embracing AI in our institution, but no one tells us how or where the boundaries are.”*

Similarly, Participant 2 abandoned a GenAI-based writing activity after her university launched a campaign on AI misuse, fearing accusations of promoting plagiarism. These reflections demonstrate how unclear institutional signals can translate into teacher anxiety, leading to behavioral restraint and the withdrawal of otherwise pedagogically sound practices.

*“After the AI ethics campaign started, I noticed many colleagues stopped mentioning GenAI at all. I had planned a writing task using AI paraphrasing tools, but I canceled it—I didn’t want to be accused of promoting plagiarism. The atmosphere made it feel like AI had no place in ethical education.”*

Structural inequalities further shaped how GenAI was incorporated into classrooms. Teachers working in institutions with uneven access to digital infrastructure described the challenges of assigning tasks that required reliable internet or personal devices. As Participant 1 stated,

*“I’d love to use GenAI tools more often, but the reality is that half my students can’t access them outside the classroom. Some don’t have laptops, some have limited data. I can’t assign a task I know only part of the class can complete. It wouldn’t be fair. So I’ve had to limit its use even though I see the benefits.”*

Such disparities illustrate how broader socioeconomic conditions mediate both student participation and teacher decision-making, limiting the equitable use of GenAI despite widespread recognition of its pedagogical benefits.

As illustrated across the three narrative levels—story, Story, and STORY—university EFL teachers were able to frame their teaching experiences related to GenAI by including personal reflections, contextual classroom practice, and wider sociopolitical contexts. These multiple narrative layers illustrated not only an individual’s beliefs and feelings about GenAI tools but also institutional practices and national policies. As each participant had a different pedagogical identity formed by earlier experiences, the participants’ engagement with GenAI tools was mediated by highly personal interpretations of what it may mean to engage students in learning. This reinforces the need for teachers to clearly establish connections between their own learning paths and the continually evolving needs of their students. In summary, engaging students cognitively, emotionally, and behaviorally with GenAI tools necessitates a deeper, reflective consideration of how psychological, interpersonal, institutional, and sociopolitical nuances interrelate—represented in the trajectories of participants’ narratives.

## 4. Discussion and Conclusions

This research aimed to investigate how EFL teachers at the university level conceptualize and incorporate GenAI tools in teaching to promote engagement (Fredricks et al., 2004; Reeve & Tseng, 2011).

Our study, framed by Barkhuizen’s (2007, 2016) three-layered narrative framework, enables meta-inferences rather than viewing each level of analysis in isolation. Specifically, it highlights how classroom practices (story), professional identities (Story), and systemic constraints (STORY) intersect to create a multi-layered and often contradictory picture of teacher engagement. For example, while story emphasized personal classroom experiences, Story captured changes in teacher identity, and STORY reflected the systemic challenges and opportunities that the action researchers faced. Taken together, these layers illustrated the multi-scalar complexity of teachers’ engagement with GenAI.

Consequently, GenAI not only prompts teachers to shift from individual experimentation toward rethinking their professional identities but also balances personal agency with institutional constraints. This change at multiple layers positions technology as not simply a tool but as a disruptor of practices and an agent of pedagogical change. Moreover, these narratives can be interpreted more fully when considered through the lenses of Self-Determination Theory (SDT), Control–Value Theory (CVT), and Teacher Cognition Theory. Together, these theories provide valuable insights into how teachers enact GenAI pedagogy while also illuminating moments of conflict and negotiation in their practice.

To be more specific, at the story or micro-level of analysis, participants described specific classroom instances in which they used and explored GenAI tools like ChatGPT to promote idea generation, scaffold conversation, or provide feedback. These moments resonate with SDT’s emphasis on competence as a fundamental need for motivation (Deci & Ryan, 2000). Teachers observed that even previously passive students became cognitively active when supported by GenAI, a finding that aligns with studies showing that AI writing tools can stimulate deeper learner agency and problem-solving (Gayed et al., 2022; Xu et al., 2025). At the same time, varied perspectives emerged: while some educators interpreted these tools as providing agency, others were concerned that reliance on AI might hinder the same competency that SDT recognizes as important. The challenge here shows that the perceived worth of cognitive engagement is not consistent but mediated by teachers’ pedagogical beliefs.

In addition to these narratives, stories at the Story level also reflected how participants’ larger professional identities and philosophies of teaching influenced their thoughts about using GenAI in practice, which is also related to behavioral engagement, especially in terms of both pedagogy and their own professionalism. Many noted their understanding of their changing role—from being “knowledge transmitters” to inquiry facilitators. This represents a direct connection to Teacher Cognition Theory, which asserts that the beliefs and experiences teachers bring to their practice are central to the construction of practice (Borg, 2006; Kubanyiova & Feryok, 2015). Others emphasized that GenAI’s use should align with pedagogical beliefs such as fostering learner autonomy, critical thinking, and meaningful communication. This suggests that technology integration was perceived not merely as a technical choice but as a pedagogical and ethical decision (Kessler, 2018; Warschauer & Xu, 2024). Moreover, participants, who had been trained in exam-focused systems, expressed a desire to move away from a restrictive model, using GenAI to allow for learner-centered, dialogic pedagogies. Others stated that they limited the use of GenAI to meet strict pedagogy so as not to leave depth behind in order to comply with the curriculum. This dichotomy provides another layer of complexity when considering the structural constraints in school that still mediate behavioral choices. It reflects what Farrell (2013) argues, in that reflective practice is often informed by autobiographical knowledge and leads to pedagogical agency.

In line with emotional engagement, participants expressed complicated feelings from curiosity and a sense of optimism to anxiety and frustration. These emotional stances correspond closely with CVT in the sense that achievement passions arise through the interaction of perceived control and task value (Pekrun, 2006). For example, when teachers felt in control (by framing GenAI as a tool for brainstorming instead of a replacement), they expressed excitement and sometimes relief at their students’ motivation. However, when the institutional uncertainty related to plagiarism led to a diminished sense of control, the teachers expressed anxiety and withdrew from innovative pursuits. These disparate emotional responses mirror contemporary scholarship showing that teachers’ emotions offer not only an important role but perhaps a defining role in the process of adopting new technologies (Zhi et al., 2023). In this way, GenAI is an emotionally empowering tool and an anxiety-inducing source of professional discomfort depending on contextual affordance and constraints.

At the macro-level (STORY), participants understood their practices to be implicated within wider institutional and policy contexts. Several teachers explained that national educational policies, centrally prescribed curricula, and top-down directions around assessment restricted their ability to adopt innovative tools such as GenAI, which demonstrates the challenges of balancing external mandates with teacher autonomy. For example, the Council of Higher Education (YÖK) published the Ethical Guidelines on the Use of Generative Artificial Intelligence in Scientific Research and Publications in 2024, emphasizing principles such as transparency, honesty, fairness, accountability, and respect when using AI tools in academic contexts (YÖK, 2024). These findings parallel earlier critiques in the literature of structural barriers to pedagogical innovation in EFL contexts (Kumaravadivelu, 2006). Importantly, these systemic pressures also had direct implications for student engagement: restrictive curricula constrained cognitive engagement by limiting inquiry by scope, reduced emotional engagement by fostering frustration or anxiety on the part of the teacher and/or students, and restricted behavioral engagement by discouraging the exploration of new learning practices.

Additionally, participants identified issues of digital inequity, which meant students’ differing levels of access to technology and abilities around digital literacy often widened the gap between potential and practice in the classroom. The unequal access did not just limit students’ behavioral engagement (for example, unequal access to technology prevents equal engagement in completing tasks) but also influenced their emotional engagement because those not in a position to acquire technology felt inadequate. This supports a growing discussion in the literature around AI and educational equity (Selwyn, 2011; Varsik & Vosberg, 2024) and suggests that the promise of GenAI must be critically interrogated with respect to social justice.

Taken together, the evidence indicates that GenAI in EFL classrooms functions not merely as a technological innovation but also as a boundary object that mediates between institutional structures and teacher agency (Zhang & Dong, 2024; Lo, 2025). Although systemic constraints (e.g., national curricula and high-stakes exams) governed the extent of teachers’ agency, they were able to creatively adapt ways to leverage GenAI to engage their students. This interplay supports Kumaravadivelu’s (2006) assertion that pedagogical innovation in EFL contexts often comes into conflict with structural constraints, but also reinforces that teachers’ agency can reshape constraints that obstruct their innovation into opportunities. Perhaps most importantly, the results present evidence that GenAI engagement is never one-dimensional: the cognitive, emotional, and behavioral dimensions co-occur in ways that embody their lived identities, or work as affective contract, and embody the institutional contexts in which they are situated.

Overall, this study adds to existing discussions by demonstrating that the acceptance of GenAI cannot simply be understood through technical affordances and ethical implications. Rather, the interplay of psychological needs (SDT), affective appraisals (CVT), and professional beliefs (Teacher Cognition) informs how educators make sense of and operationalize new technological possibilities. While this analysis underlines teacher narratives, it also makes clear that integrating GenAI develops opportunities for novel pedagogic practice at the same time as potential dilemmas regarding professional integrity. Although this may be complicated and concerning to educators, it also facilitates GenAI’s role in expanding the generative opportunities for reflection, negotiation, and transformation within EFL pedagogic practice.

The significant implication of this study is that integrating GenAI should not be understood solely in terms of technological literacy or novelty but rather as educators engaging in critical pedagogical reflection and contextual awareness. Teacher education programs need to go beyond technology training that frames teacher education as an additive approach and incorporate reflective, narrative engagement that supports educators in interrogating their assumptions, institutional constraints, and ethical responsibility (Farrell, 2013; Barkhuizen, 2017). By introducing educators to wide-ranging reflective practices regarding their experiences with GenAI, professional development opportunities can stimulate deeper critical inquiry and facilitate adaptive and context-sensitive uses of technology in language classrooms.

Regarding the limitations of this study, although the results provide an important overview of EFL educators’ experiences with GenAI tools. The generalizability of the study is restricted to the geographical and institutional background of the participants. All of the participants were from the same institutional and higher education framework in Türkiye. Thus, the results should not be regarded as statistically generalizable but rather as transferable to similar contexts in higher education, in keeping with qualitative research tradition. Although narrative inquiry does not seek statistical generalizability but instead contextualized understanding and theoretical transferability (Clandinin & Connelly, 2000), future research might engage with the limitations of this study in a cross-national comparative study. In this way, it can analyze the EFL educator’s integration of GenAI professional practices, agency, and motivation to implement GenAI tools for EFL learning in various sociopolitical, cultural, and educational contexts. In addition, it would expand our understanding of the structural and contextual conditions of teacher cognition, professional experience, and technology interactions within these global contexts. Longitudinal studies would provide insights into how educators’ use of GenAI tools develops over time, particularly in response to changes in technology or policy. Furthermore, including student perspectives would enhance our understanding of GenAI’s influence on engagement across cognitive, emotional, and behavioral aspects. These studies would provide a more holistic view of how GenAI is negotiated in different EFL contexts and help foster more equitable and context-sensitive ways to integrate technology. Comparative studies might also study a wide array of issues related to institutional context, EFL educator training, and geographical regions to understand how every issue mediates perception and integration of GenAI tools. Finally, future studies should consider students’ perspectives to understand the overall experience of GenAI in the classroom more collaboratively. This study contributes to existing knowledge and understanding by providing a narrative–inquiry study that EFL educators in higher education can interpret to enact GenAI tools to promote student engagement. By centering the narratives of educators, this study seeks to privilege the lived experience of EFL teachers instead of the centralized EdTech narrative that privileges technical affordances and learning outcomes as an indication of successful engagement (Zawacki-Richter et al., 2019). This study also did not engage in systematic analyses of the formal curriculum, the hidden curriculum, or co-/extra-curricular spaces, but it is suggested that these analyses be pursued as a path forward for future research.

All in all, the findings of this study highlight a need for educational policies—especially in the context of Turkish higher education—that recognize that GenAI still exists in classrooms and empowers teachers to use new tools in ways that are consistent with local values and learner needs, as well as their professional ethics. As our findings illustrate, EFL teachers’ ability to implement GenAI meaningfully is shaped not just by their pedagogical beliefs but by their degree of institutional support and autonomy. Policymakers and educational leaders must move beyond generalized calls for digital transformation and instead develop clear, inclusive guidelines that prioritize teacher training, provide ethical frameworks, and recognize the diverse contexts in which educators operate. In particular, promoting teacher autonomy—by allowing for space for professional judgment, experimentation, and adaptation—is essential for fostering innovation without compromising pedagogical integrity. Without such structural support—at least in the present Turkish context—even the most enthusiastic and capable teachers may disengage from GenAI integration due to institutional ambiguity or risk aversion.

## Figures and Tables

**Table 1:** Participants’ demographic information.

Participants	Gender	Undergraduate Department	Years of Experience	Familiarity with GenAI Tools
P1	Male	ELT	3 years	Moderate
P2	Female	Translation and Interpretation	8 years	High
P3	Female	ELT	12 years	High
P4	Male	Literature	20 years	Moderate
P5	Female	Literature	5 years	Low
P6	Male	Translation and Interpretation	15 years	Low
P7	Male	ELT	10 years	Moderate
P8	Female	ELT	6 years	High
P9	Female	ELT	22 years	Low

**Table 2:** Summary of narrative levels, key themes, and representative quotes.

Narrative Level	Key Themes	Representative Quotes
story (individual experiences)	Navigating uncertainty; experimenting with GenAI	“At first, I wasn’t sure how to use it effectively, but then I realized it could help me prepare activities faster.” (P2)
Story (professional identity and pedagogical beliefs)	Shifting teacher roles; aligning with autonomy and critical thinking	“I see myself less as the provider of knowledge, more as a facilitator of student inquiry.” (P4)
STORY (broader institutional and social context)	Negotiating policy constraints; empowering underrepresented voices	“It felt like the tool had given her a voice.” (P3)

## Data Availability

The data that support the findings of this study are available from the corresponding author upon reasonable request.

## Abbreviations

The following abbreviations are used in this manuscript:

GenAI	Generative Artificial Intelligence
EFL	English as a Foreign Language
ELT	English Language Teaching
EdTec	Educational Technology
CVT	Control–Value Theory
